# Salinity and Temperature Dual-Parameter Sensor Based on Fiber Ring Laser with Tapered Side-Hole Fiber Embedded in Sagnac Interferometer

**DOI:** 10.3390/s22218533

**Published:** 2022-11-05

**Authors:** Fang Zhao, Weihao Lin, Jie Hu, Shuaiqi Liu, Feihong Yu, Xingwei Chen, Guoqing Wang, Perry Ping Shum, Liyang Shao

**Affiliations:** 1 Department of Electrical and Electronic Engineering, Southern University of Science and Technology, Shenzhen 518055, China; 2 State Key Laboratory of Analog and Mixed-Signal VLSI, University of Macau, Macau 999078, China; 3 School of Microelectronics, Shenzhen Institute of Information Technology, Shenzhen 518172, China; 4 Peng Cheng Laboratory, Shenzhen 518005, China

**Keywords:** salinity sensor, tapered dual-side-hole fiber, Sagnac interferometer

## Abstract

This paper presented a new kind of salinity and temperature dual-parameter sensor based on a fiber ring laser (FRL) with tapered side-hole fiber (SHF) embedded in a Sagnac interferometer. The sensing structure is majorly composed of tapered SHF located in the middle of SHF inside the Sagnac interferometer loop structure. The influences of the SHF’s diameters of different tapered in the Sagnac interferometer loop on the FRL sensing system are studied. The presence of air holes in the SHF makes the cladding mode easier to excite, and the interaction between the cladding mode with its surroundings is enhanced, thus having higher salinity sensitivity. Besides, the unique advantages of high resolution, narrower linewidth, and high signal-to-noise ratio (SNR) of fiber laser make the measurement results more accurate. In this experiment, the SHF with different taper diameters was made, and it was found that reducing the diameter of the taper waist diameter could further improve the salinity sensitivity. When the waist diameter was 9.70 μm, the maximum salinity sensitivity of 0.2867 nm/‰ was achieved. Temperature sensing experiments were also carried out. The maximum temperature sensitivity of the FRL sensing system was −0.3041 nm/°C at the temperature range from 20 to 30 °C. The sensor has the characteristics of easy manufacture, good selectivity, and high sensitivity, proving the feasibility of simultaneous measurement of seawater salinity and temperature.

## 1. Introduction

Salinity is an important standard for the quantitative measurement of seawater and one of oceanography’s most important physical and chemical parameters. It is closely related to coastal runoff, precipitation, and sea surface evaporation. The distribution and change of salinity are also important factors that affect and restrict the distribution and change of other hydrological elements [1,2]. Therefore, the accurate measurement of seawater salinity is important for marine hydrological observation.

Fiber optic technology for salinity measurement has recently attracted extensive attention due to the advantages of high sensitivity, low cost, anti-electromagnetic interference, and strong stability [3]. In the last few years, various fiber optic salinity sensors have been proposed, including fiber Bragg grating (FBG) [4], Fabry–Perot interferometer [5], MZI [6,7,8], Sagnac interference loop [9], surface plasmon resonance (SPR) [2], etc. Because different sensing structures have distinctive advantages, combining different methods can give full play to the advantages of the whole sensing system and has become a new research hotspot.

Although most fiber sensors show good sensing performance, there are still some shortcomings, such as low sensitivity and manufacturing technology. For example, Pereira et al. performed simultaneous temperature and salinity measurements by cascading two FBGS, one of which was chemically corroded [10]. D. Luo et al. presented a fiber optic salinity and temperature sensor coated with a polyimide layer on etched fiber grating with sensitivities of 0.025 nm/‰ and 0.043 nm/°C, respectively [11]. M. Sun et al. coated lamellar polyimide on FBG with a salinity sensitivity of 0.0358 nm/% and temperature sensitivity of 0.0321 nm/°C [12], which is two times more than Men et al. realized [13]. However, the low sensitivity limits the use of sensors in high-precision scenarios.

JC Zhao et al. fabricated a fiber sensor using SMF-MMF structure lateral offset connected with UV-coated photonic crystal fiber. Its salinity sensitivity is 2.495 nm/‰, and the temperature sensitivity is 1.486 nm/°C [14]. J. Zhao et al. designed a hollow-core fiber (HCF) cascade no-core fiber (NCF) structure sensor. HCF has a laser-carved u-shaped groove and NCF-coated polymer for temperature measurement. The salinity and temperature experiments show that the sensitivity is 0.235 nm/‰–4.948 nm/°C, respectively [15]. S. Wang et al. demonstrated a Hybrid MZI through silica and fluorinated polyimide microfibers. The salinity and temperature experiments show that the sensitivity is 0.064 nm/‰ 0.14 nm/°C, respectively [16]. But the fabrication process of the above structures is complicated because the fibers need to be coated or encapsulated with polymers, which also limits sensor stability and long-term use.

Y. Zhao et al. designed a fiber optic sensor with cascades of the Fabry–Perot interferometer (FPI) and MZI using femtosecond laser etching in a hollow fiber. The sensitivity of the salinity is 0.244 nm/‰, and the sensitivity of temperature is 2.767 nm/°C [17]. But femtosecond laser equipment is expensive. Although some of the optical fiber interferometers above have high sensitivity in measuring salinity through spectral drift, they still have certain defects in signal-to-noise ratio (SNR), corresponding bandwidth, and production complexity.

In this paper, an FRL-based sensing system for salinity and temperature measurement is proposed and experimentally demonstrated. The sensing part is based on a tapered SHF embedded in a Sagnac interferometer loop. In this system, the sensor acts as a salinity and temperature-sensing component and simultaneously plays the role of the filter for laser wavelength selection. The change in the external environment will offset the filter’s interference spectrum, leading to the output laser wavelength changing with the environmental disturbance. The presence of air holes in the SHF makes the cladding mode more easily excited and enhances the interaction between the cladding mode and the surrounding environment, resulting in higher salinity sensitivity. By comparing the sensing experiment in the FRL with that in the broadband light source (BBS), the spectrum of the laser has high evanescent radio (ER), high-quality factor (Q), narrower linewidth, hence the excellent characteristics of the laser can improve the resolution, power, and SNR of the detection spectrum, which is suitable for high-resolution and remote measurement applications. The proposed sensing system has potentially significant value for marine environmental monitoring and high refractive index sensing applications such as chemical or biological.

## 2. Sensing Principle and Fabrication

### 2.1. Sensing Principle of the Sensor

**Figure 1:**
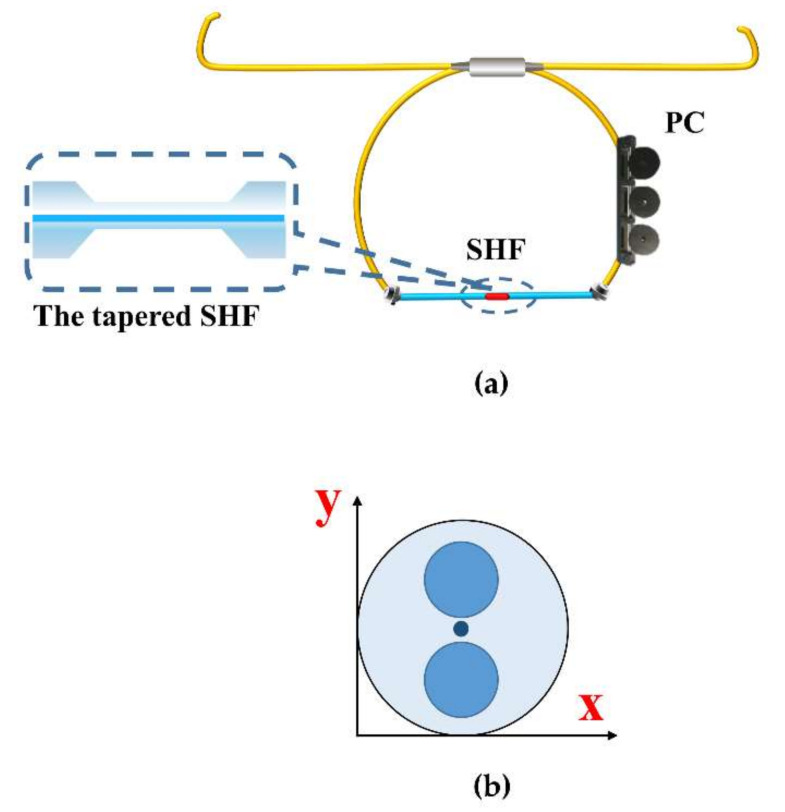
(**a**) Sagnac interferometer embedded tapered SHF structure. (**b**) Cross-sectional schematic of SHF.

The schematic layout of the sensor is shown in Figure 1a. The Sagnac interferometer loop embeds a section of 30 cm length SHF fiber with a tapered structure in the middle of it. The incident light splits into two beams at the 3 dB coupler, which travel clockwise and counterclockwise. In both directions, when the basic mode of SMF passes through the down-taper region of the MZI structure, part of the light propagates in the fiber core, and part of the light is coupled to the cladding and turn-to-be cladding modes. The light in the cladding in contact with the environment to sense the surrounding environment changes. Then, when the light passes through the up-taper region, the core and cladding modes re-enter the core of the SHF and interact with each other inside the fiber.

The cross-section of the SHF (Yangtze Optical Electronic Co., Ltd., Wuhan, China) is shown in Figure 1b. The core and cladding diameters are 9 μm and 114 μm, respectively. Two air holes with a diameter of 37 μm are symmetrically distributed, and the distance between the air holes and the edge is 12 μm. The large air holes of SHF have high birefringence performance and core mode confinement ability [18]. Consequently, the existence of air holes can make the cladding mode very easy to excite and make the cladding mode more sensitive to external salinity.

Assuming that the injected light intensity by introducing the fiber is $I_{\mathrm{in}}$, and ignoring the fiber insertion loss, the output intensity I can be expressed as [19,20]:

(1) $$I=\frac{I_{\mathrm{in}}(1-\cos\varphi )}{2}$$

The phase difference between clockwise and counterclockwise beams φ can be expressed as [21,22]:

(2) $$\varphi =\frac{2\pi \mathrm{BL}}{\lambda }$$

λ is the wavelength of the light, *B* is the birefringence, *L* is the effective interaction length of the SHF, λ is the wavelength. Birefringence B represents the effective refractive index difference between the two orthogonal polarization fundamental modes $\left|n_{x}-n_{y}\right|$.

As thermal expansion and thermo-optic effects exist in optical fibers, when the temperature changes, the volume change in the two orthogonal directions is not the same, and so is the equivalent refractive index variation, due to the asymmetric structure. Moreover, due to the asymmetric structure, there is a smaller volume of quartz in the polarization direction with air holes (vertical direction in Figure 1b). When the external refractive index changes, the equivalent refractive index of this polarization direction is more likely to change, resulting in the inconsistency of the effective refractive index change in the two orthogonal directions. Thus, the B changes, which is the basis of this sensing system [23].

The SHFs were tapered to different diameters, as shown in Table 1. The tapered waist diameters of S1, S2, and S3 are 7.9 μm, 11.63 μm, and 18.21 μm, respectively. The taper distance of each sensor is 2.4 cm, 2.2 cm, and 1.6 cm, respectively.

Tapered fiber can improve the sensing sensitivity. According to Equation (2), parameters B and L play a key role in sensitivity. When the fiber becomes thinner, the volume per unit length becomes smaller, and the change in the external refractive index or temperature is more likely to amplify the inconsistency of the effective refractive index changes in the two orthogonal directions. Besides, when the fiber is tapered, the sensing length becomes longer because the total amount of quartz is fixed. Tapered fiber amplifies the variation of parameter B and sensing length L so that sensitivity can be enhanced.

### 2.2. Fabrication of the Structure

The fabrication process is divided into two steps: fiber splicing and fiber tapering. Firstly, a section of the SHF with a length of 30 cm is fusion spliced with two single-mode fibers of a 3 dB coupler. It is worth noting that, in the process of fusion splicing, the splicing mode is adjusted to manual splicing mode to avoid hole collapse by reducing power and time. The parameters used in the experiment are as follows: fiber prefusion power is −50 bit, prefusion time is 10 ms, discharge power is standard −50 bit, and discharge time is 500 ms. The midpoint O is marked in the midpoint of SHF. The coating layer of the SHF is removed symmetrically on both sides of midpoint O. Install it on the optical microfiber tapering equipment, and the middle point O is placed under the fire position. The fiber optic tapering machine used in the experiment is manufactured by Shandong Coupler Technology Co., Ltd., Shandong, China (AFBT-8000LE-H0). The tapering speed is set to 500 μm/S, and the heating time is 80s.

During the tapering process, the Sagnac interferometer loop is connected to the BBS. The transmission spectrum is monitored by an optical spectrum analyzer (OSA) in real-time to ensure the stable transmission of the optical polarization in the fiber Sagnac interferometer loop. By observing the output spectrum of the OSA (AQ6370D, YOKOGAWA), it can be found that the waist diameter has a direct impact on the interference intensity loss of the Sagnac interferometer loop. The thinner the tapered waist, the greater the loss of interference spectral intensity.

## 3. Experimental Results and Discussion

### 3.1. Experimental System

The Sagnac interferometer loop consists of a 2 × 2 port 3-dB fiber coupler with a split ratio of 50:50, consisting of a section of SHF with a length of 30 cm tapered inside and a polarization controller (PC). The incident light is divided into two beams by the 3 dB fiber coupler, which travels clockwise and counterclockwise, respectively, and forms interference in the coupling region.

### 3.2. Salinity Response Based on BBS

**Figure 2:**
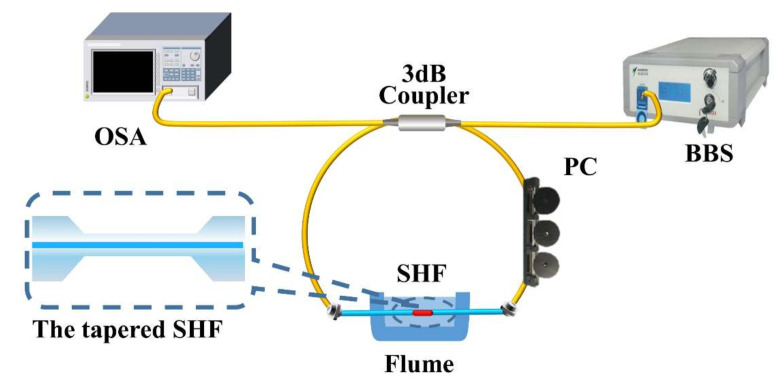
BBS sensing system, inset: tapered SHF.

Firstly, we analyzed the salinity sensing property of the proposed sample S1 under BBS. As shown in Figure 2, two ends of the 3 dB coupler are connected with a BBS and OSA, respectively, to test the structure’s sensitivity.

**Figure 3:**
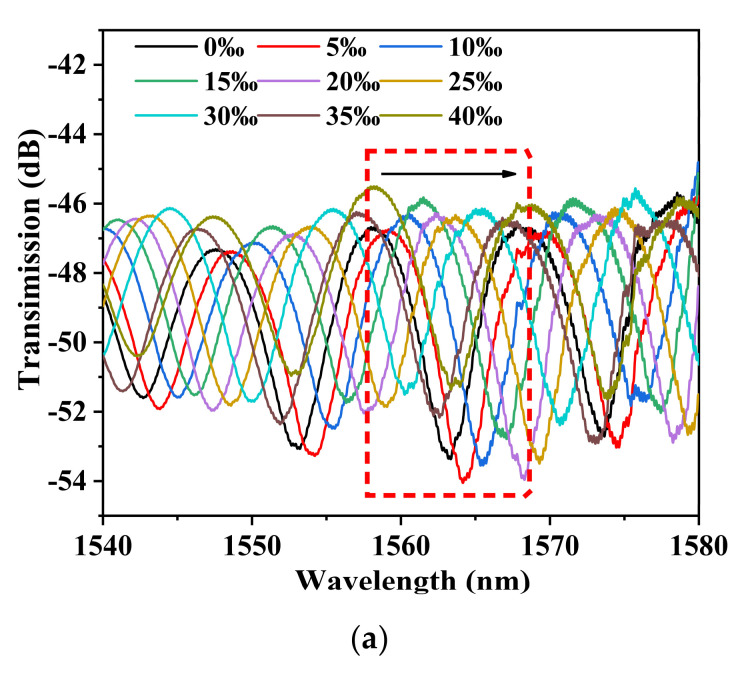

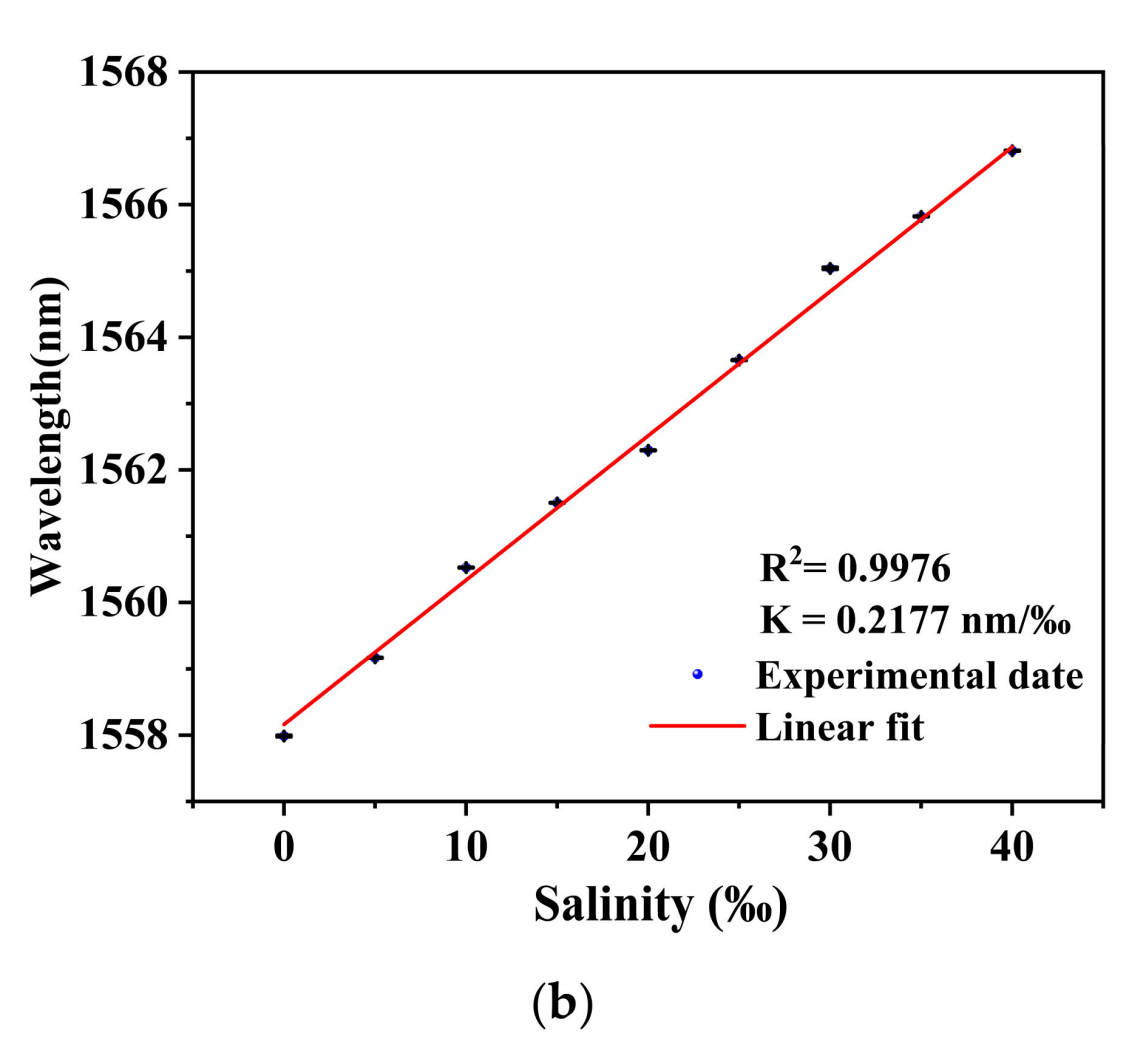
(**a**) Transmission spectrum of sensor S1 at different salinities under a broadband light source. (**b**) Relationship between resonance wavelength and salinities.

To facilitate the comparison of sensitivity under different light sources, the wavelength shifts of the peak near 1550 nm are selected to study the salinity sensing characteristic. Experimental results of the salinity sensitivity of sample S1 under BBS are shown in Figure 3. When the salinity of the external environment increases from 0‰ to 40‰, with an interval of 5‰, the sensitivity of the salinity measurement is 0.2177 nm/‰, and the linearity is 0.9976.

**Figure 4:**
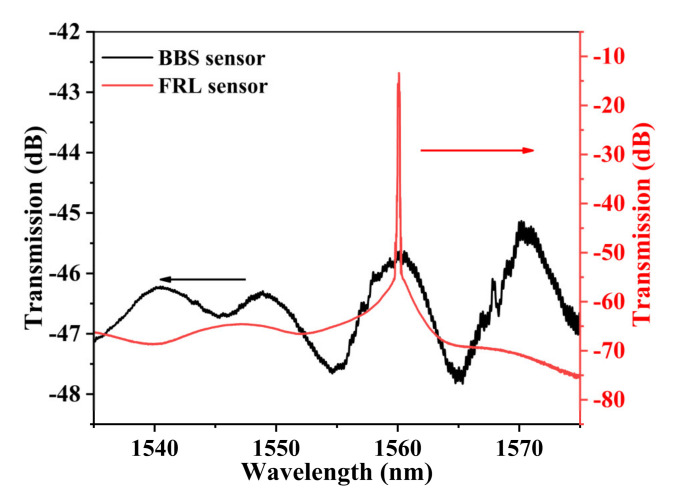
Transmission spectrum of sensor S1 at the same temperatures under BBS (black line) and FRL (red line).

Figure 4 shows the transmission spectrum of sensor S1 at the same temperature under BBS (black line) and FRL (red line). The FRL output spectrum is in good agreement with the BBS. The output laser wavelength of FRL is mainly determined by the filtering characteristics. The red curve is the output spectrum of the fiber laser sensing system with an emission wavelength of about 1550 nm, and the threshold pump power of the 980 nm semiconductor pump source is about 400 mW.

The optical fiber sensing technology based on an optical FRL cavity can obtain the physical quantity changes of the external environment by using the wavelength shift caused by the filter modulation in the optical fiber transmission process. It provides a new demodulation scheme for point fiber sensing because of the advantages of a high SNR, narrow 3-dB bandwidth, and high Q [24].

### 3.3. Salinity Response Based on Fiber Ring Laser

**Figure 5:**
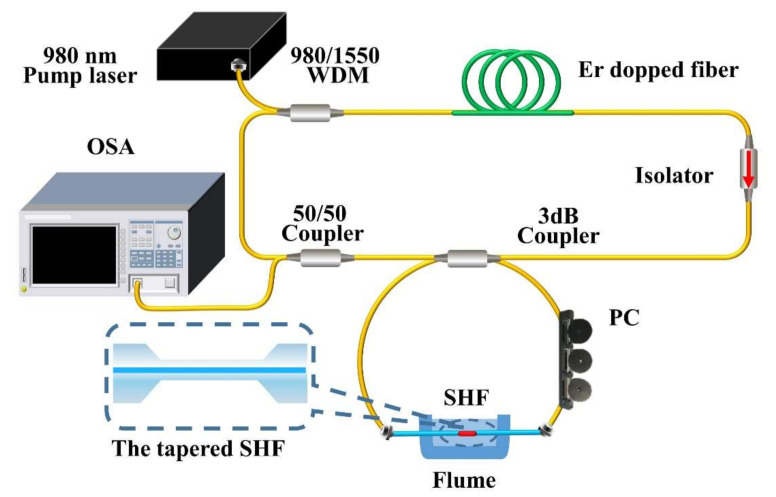
Fiber ring laser sensing system, inset: taper SHF.

To improve the comprehensive sensing performance of the sensor, an optical FRL sensor based on Sagnac interferometer embedded MZI is proposed and experimentally investigated, as shown in Figure 5. The sensor can effectively enhance the visibility of the response spectrum while reducing the corresponding 3-dB bandwidth. The principle of the designed FRL system is shown in Figure 5. The system uses pump light with a central wavelength of 980 nm, which is coupled into the laser cavity through a 980/1550 wavelength division multiplexer (WDM). The operating threshold of the pump light is 400 mW. An Erbium-doped fiber (Fibercore Ltd. model 1-15(980/125) HC) with a length of 220 cm is used as the gain medium. The C-band isolator ensures the unidirectional cyclic transmission of optical light in the laser ring cavity.

Since the Sagnac interferometer loop embedded tapered SHF structure can form an interference spectrum, it can be inserted into the resonator of the laser to work as a filter in the laser cavity. A 50/50 fiber coupler was used to separate 50% of the light energy in the laser cavity. A part of the light was split for cycle amplification of the light energy, while the others separated from the cavity were emitted into the OSA for spectrum analysis. Before adding the salt solution, we maximized the output power of the laser by adjusting the PC to ensure that the laser worked in the optimal state. A series of experiments were implemented to study the sensing characteristics of the FRL sensor and prove the superiority of its sensing ability. During the experiments, the sensor head of the optical fiber laser system was placed in the water tank. Then, we separately added salt solutions with mass fractions ranging from 0‰ to 40‰ to the water tank. The laser output wavelength varies with salinity due to the change of polarization state in the Sagnac interferometer loop.

The experiment adopts the control variable method. Keep the salinity constant when measuring temperature changes. To further avoid salinity changes caused by evaporation during the experiment, the tank was filled with deionized water for the experiment. To avoid the influence of temperature, bending, and other environments during the experiment, the water tank and sensing structure are placed in the thermostat during the experiment, and the SHF fiber is placed horizontally. The spectrum is recorded after the temperature is stable. When measuring salinity changes, keep the sensor structure horizontally in the thermostat, and change different concentrations of the solution. In each set of experiments, the results are recorded at 3 min, 5 min, and 7 min, respectively, and the data of the three experiments are processed by the error bar.

In general, the salinity of seawater around the world ranges from 0 to 35‰ [25]. The experiment used 0~40‰, covering the salinity range of seawater. The temperature range of 20 to 32 °C, covers the range of seawater temperature measurements in many ocean regions [17]. However, due to the mode hopping characteristic of the laser itself, the measurement range is set to 20–30 °C.

Furthermore, we studied the influence of different diameters of tapered SHF on salinity and temperature sensitivity. Salinity sensing experiments with different diameters were in progress. As shown in Table 1, the tapered waist diameters of S1, S2, and S3 are 7.9 μm, 11.63 μm, and 18.21 μm, respectively. The taper distance of each sensor is 2.4 cm, 2.2 cm, and 1.6 cm, respectively.

**Figure 6:**
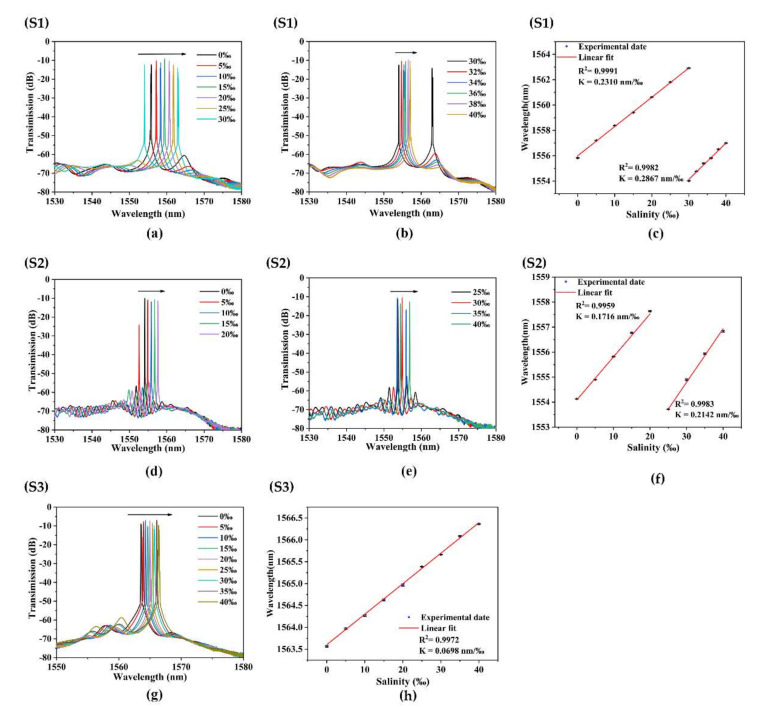
Salinity measurement results of S1, S2, and S3. (**a**,**b**,**d**,**e**,**g**) Wavelength shift with increasing salinity of each sensor. (**c**,**f**,**h**) Wavelengths response to salinities of each sensor.

Figure 6 shows that when the salinity of the external environment increases from 0‰ to 40‰, the transmission spectrum shifts to a long wavelength. The salinity experiment results of the S1 sensor are shown in Figure 6a–c. The corresponding 3-dB bandwidth is 0.04 nm. The visibility of the interference SNR is 58.96 dB. When the salinity of the external environment increases from 0‰ to 30‰, with an interval of 5‰, the sensitivity of salinity measurement is 0.2310 nm/‰, and the linearity is 0.9991. When the salinity of the external environment increases from 30‰ to 40‰, with an interval of 2‰, the sensitivity of salinity measurement is 0.2867 nm/‰, and the linearity is 0.9982.

The resolution of the spectrometer used in the experiment is 0.02 nm. Thus, the max salinity sensor has a resolution of 0.070‰. The Sagnac interferometer loop plays the role of sensor and filter. The laser output is near the maximum peak of the interference peak. When the salinity offset is too large, the laser pulse cannot transition to the next wavelength. After mode competition, lasers of different wavelengths continue to output laser at the original position (around 1550 nm). Therefore, the detection range of the sensor should be divided into two parts (0–30‰; 30–40‰).

The salinity experiment results of the S2 sensor are shown in Figure 6d–f. The corresponding 3-dB bandwidth is 0.04 nm. The visibility of the interference SNR is 59.637 dB. When the salinity of the external environment increases from 0‰ to 20‰, with an interval of 5‰, the sensitivity of salinity measurement is 0.1716 nm/‰, and the linearity is 0.9959. When the salinity of the external environment increases from 25‰ to 40‰, with an interval of 5‰, the sensitivity of salinity measurement is 0.2142 nm/‰, and the linearity is 0.9983.

We have carried out experimental research on S3 sensors based on the same method. The salinity experiment results of the S3 sensor are shown in Figure 6g,h. The corresponding 3-dB bandwidth is 0.04 nm. The visibility of the interference SNR is 59.273 dB. The salinity sensitivity of sample S3 is 0.0698 nm/‰, and the linearity is 0.9972. It can be seen from Figure 6 that the transmission spectrum of FRL’s wavelength shifts linearly with increasing water salinity.

### 3.4. Temperature Response Based on Fiber Ring Laser

**Figure 7:**
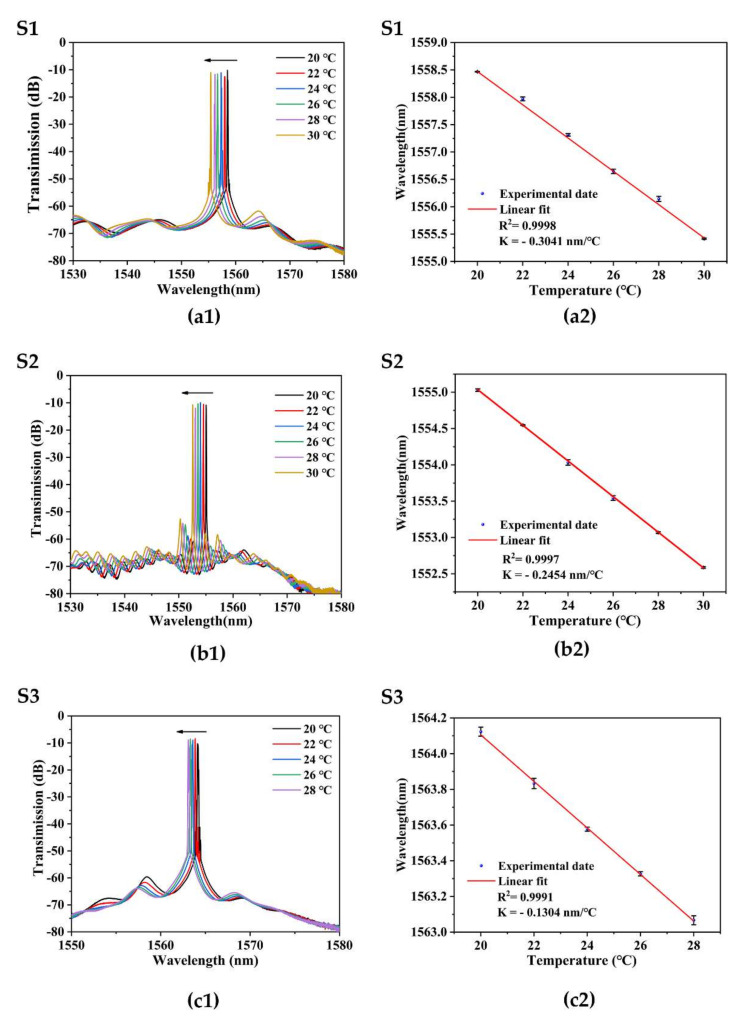
Temperature measurement results of S1, S2, and S2. (**a1**,**b1**,**c1**) Wavelength shifts with increasing temperatures of each sensor. (**a2**,**b2**,**c2**) Wavelengths response to temperatures of each sensor.

The temperature experiment result of each sensor is shown in Figure 7. When the temperature of the external environment increases, the transmission spectrum of FRL shifts to a short wavelength. Figure 7a1,a2 shows that the temperature sensitivity of sample S1 is −0.3041 nm/°C, and the linearity is 0.9998. The temperature experiment result of the S2 sensor is shown in Figure 7b1,b2. When the temperature increases from 20 °C to 30 °C with an interval of 2 °C, the sensitivity of sample S2 is −0.2454 nm/°C, and the linearity is 0.9997. The output temperature result of the S3 sensor is shown in Figure 7c1,c2. When the temperature increases from 20 °C to 28 °C with an interval of 2 °C, the temperature sensitivity of sample S3 is −0.1304 nm/%, and the linearity is 0.9991.

### 3.5. Discussion

Table 2 compares the experimental conclusions of sample S1, S2, and S3 sensors with different fiber diameters of tapered SHF. By comparison, it can be concluded that the sensitivity of the salinity of the sensor increases with the decrease of the taper diameter. Equation (2) can well explain this phenomenon. The explanation is that the tapered fiber amplifies the variation of parameter B and sensing length L, with the increase of the salt solution concentration. The effective interaction length L of S1 is the longest, and the phase difference between the clockwise and counterclockwise beams increases accordingly. For this reason, the sensitivity of the sensor is increased.

It can be concluded from Table 2 that the temperature sensitivity of S1 is higher than that of S2 and S3. When the temperature changes, due to the asymmetric structure of the fiber, the thermal expansion effect and the thermal optical effect of the fiber and the air are different, and the volume changes in the two orthogonal directions are not the same, and the equivalent refractive index changes are also different. Therefore, the diameter of the taper becomes smaller, and the effective interaction length L becomes longer, so the value B becomes larger, thus improving the sensitivity.

According to the comparison of the changes of different sensor wavelengths with the increase of salinity of each sensor in Figure 6, it can be found that S1 has high sensitivity and relatively large wavelength movement within the same salinity range, so the mode hopping phenomenon occurs when the salinity is 30‰. Different salinity ranges of S1 are shown in Figure 6a,b. S2 also experienced mode hopping at the salinity of 25‰. The different salinity ranges of S2 are shown in Figure 6d,e. However, the sensitivity of S3 is low, and the wavelength movement in the range of 0–40‰ is small, so no mode hopping occurs. The laser output is near the maximum peak of the interference peak. When the wavelength offset is too large, the laser pulse cannot transition to the next wavelength. This triggers a mode switch. Therefore, the laser fiber sensor has a discontinuous test range in the experiment.

The sensor can be improved with the following mechanistic details: in the link of setting tapering parameters, the quality of tapering SMF can be improved by heating time, tapering speed, and so on. Although the sensitivity increases with the decrease of the diameter of the taper region, the SNR will be sacrificed, and the stability and robustness will be reduced as the diameter is reduced. Therefore, the finest tapered waist diameter used in the experiment is 7.9 μm. In addition, coating materials with high expansion rates and fast reaction times can also be combined to increase the sensitivity of sensors.

**Figure 8:**
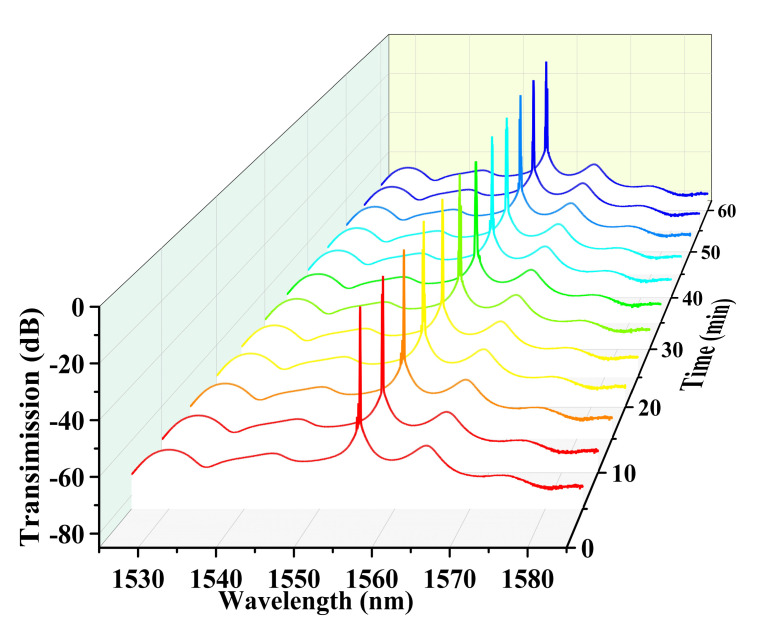
Stability test of the fiber ring laser of S1 at a constant temperature of 30 °C.

The stability of the laser emission spectrum is shown in Figure 8. The sensing head of S1 is placed in thermostatic water and records the laser spectrum every 5 min within 1 h. The figure shows that the laser wavelength change is only 0.09 nm.

The sensitivity comparison between the proposed FRL sensor and other salinity sensors in previous work is shown in Table 3. Compared with other salinity temperature sensors, our proposed salinity sensor has higher sensitivity and relatively sensitive temperature sensitivity [26], showing better potential. In addition, other works use BBS as input, so the output spectral linewidth is wide. As a comparison, the superior laser characteristics significantly improve the sensor’s comprehensive sensing performance, which can improve the resolution and SNR of the detection spectrum [27,28].

Similar to other fiber optic sensors of salinity and temperature, the phenomenon of cross-influence of temperature and salinity is also the focus of future research. Due to the laser generation mechanism, when the measurement range of the fiber laser sensor is too large, the phenomenon of mode hopping will occur. Therefore, there is a measurement interval for laser sensing. Considering that the real measured environment is relatively harsh, how to encapsulate the optical sensor to ensure the system’s accuracy, stability, and long-term use is the main direction of practical research in the future [25,29]. 

## 4. Conclusions

In conclusion, a new kind of salinity and temperature dual-parameter sensor based on FLR with tapered SHF embedded in a Sagnac interferometer is proposed and experimentally. Due to the effect of the air holes in the SHF, more light is coupled into the cladding mode, which improves salinity sensitivity. In addition, the birefringence of the tapered SHF in Sagnac also improves the temperature sensitivity. Through experimental measurement, the salinity sensitivity of the sensor reaches 0.2310 nm/‰ in the range of 0–25‰ and reaches 0.2867 nm/‰ in the range of 25–40‰. The sensor has the advantages of high resolution, narrow bandwidth, and superior optical signal-to-noise ratio of detection spectrum, which makes the FRL sensor system suitable for high-resolution or remote salinity measurement applications. Temperature sensing experiments in the FRL cavity were also carried out, and the max temperature sensitivity of the sensor reached −0.3041 nm/°C in the temperature range of 20–30 °C. Further experimental results show that the salinity and temperature sensitivity of the tapered SHF with a diameter of 7.90 μm and length of 2.4 cm are higher than those of other sizes. Own to high sensing comprehensive quality, high SNR, high sensitivity, anti-electromagnetic interference, low cost, and suitability for long-distance transmission, the proposed FRL system has potential value in ocean environment detection, industrial, and fundamental research area.

## Figures and Tables

**Table 1:** Parameters of different sensors.

Sample NO.	Waist Diameter (μm)	Taper Distance (cm)	Microscope Image
S1	7.90	2.4	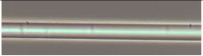
S2	11.63	2.2	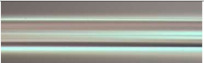
S3	18.21	1.6	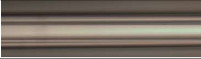

**Table 2:** Summary of the experimental results of each sensor.

Sample	Light Source	Salinity Sensitivity (nm/‰)	Salinity Range (‰)	Temperature Sensitivity (nm/°C)	Temperature Range (°C)
S1	FRL	0.2310	0–25	0.3041	20–30
		0.2867	25–40		
	BBS	0.2177	0–40		
S2	FRL	0.1716	0–20	0.2454	20–30
		0.2142	25–40		
S3	FRL	0.0698	0–40	0.1304	20–28

**Table 3:** Comparison of experimental sensitivities of different structures for salinity and temperature measurement.

Structures	Salinity Sensitivity (nm/‰)	Temperature Sensitivity (nm/°C)	Ref.
Microfiber knot resonator (2015)	0.0022	/	[30]
Polyimide layer on etched fiber grating (2017)	0.025	0.043	[11]
Optical Microfiber Coupler (2019)	0.1596	2.326	[31]
Dual-core PCF (2020)	0.2000	1.000	[32]
SPR (2020)	0.31	2.02	[33]
Balloon-shaped and core diameter mismatch (2021)	0.0168	0.7356	[34]
Sagnac interferometer concatenated PMF tapers (2021)	0.0367	0.728	[9]
Lateral offset connected with UV-coated photonic crystal fiber (2021)	2.495	1.48	[16]
FPI and MZ cascaded (2022)	0.244	2.767	[17]
This work	0.2867	0.3041	

## Data Availability

Not applicable.
